# Noise-assisted multivariate empirical mode decomposition for multichannel EMG signals

**DOI:** 10.1186/s12938-017-0397-9

**Published:** 2017-08-23

**Authors:** Yi Zhang, Peng Xu, Peiyang Li, Keyi Duan, Yuexin Wen, Qin Yang, Tao Zhang, Dezhong Yao

**Affiliations:** 1 School of Aeronautics and Astronautics, University of Electronic Science and Technology of China, Chengdu, 611731 China; 2 Key Laboratory for NeuroInformation of Ministry of Education, School of Life Science and Technology, University of Electronic Science and Technology of China, No. 4, Section 2 of North Jianshe Road, Chengdu, 610054 China; 3 Center for Information in BioMedicine, University of Electronic Science and Technology of China, No. 4, Section 2 of North Jianshe Road, 610054 Chengdu, China

**Keywords:** EEMD, MEMD, NA-MEMD, Mode-alignment, Mode-mixing, Multichannel EMG signals

## Abstract

**Background:**

Ensemble Empirical Mode Decomposition (EEMD) has been popularised for single-channel Electromyography (EMG) signal processing as it can effectively extract the temporal information of the EMG time series. However, few papers examine the temporal and spatial characteristics across multiple muscle groups in relation to multichannel EMG signals.

**Experiment:**

The experimental data was obtained from the Center for Machine Learning and Intelligent Systems, University of California Irvine (UCI). The data was donated by the Nueva Granada Military University and the Technopark node Manizales in Colombia. The databases of 11 male subjects from the healthy group were taken into the study. The subjects undergo three exercise programs, leg extension from a sitting position (sitting), flexion of the leg up (standing), and gait (walking), while four electrodes were placed on biceps femoris (BF), vastus medialis (VM), rectus femoris (RF), and semitendinosus (ST).

**Methods:**

Based on the experimental data, a comparative study is provided by assessing the Empirical Mode Decomposition (EMD)-based approaches, EEMD, Multivariate EMD (MEMD), and Noise-Assisted MEMD (NA-MEMD). The outcomes from these approaches are then quantitatively estimated on the basis of three criterions, the number of Intrinsic Mode Functions (IMFs), mode-alignment and mode-mixing.

**Results:**

Both MEMD and NA-MEMD methods (except EEMD) can guarantee equal numbers of IMFs. For mode-alignment and mode-mixing, NA-MEMD is optimal compared with MEMD and EEMD, and MEMD is merely better than EEMD.

**Conclusions:**

This study proposes the NA-MEMD approach for multichannel EMG signal processing. This finding implies that NA-MEMD is effective for simultaneously analysing IMFs based frequency bands. It has a vital clinical implication in exploring the neuromuscular patterns that enable the multiple muscle groups to coordinate while performing the functional activities of daily living.

## Background

Electromyography (EMG) is the collective electric manifestation during muscle contraction, and indicates the electrophysiological responses of motor units from a muscle group, which is controlled by the nervous system. The surface EMG signal, originating in motor units and then recorded by measurement tools, was often contaminated by various types of noises or artifacts, e.g., power line interference, baseline wandering, electrocardiographic (ECG) artifacts, capacitive effects of the detection site, and the firing rate of motor units [1–6]. Therefore, the identity of an actual EMG still remains difficult [7–9].

Recently, several methods have been developed to analyse and de-noise the EMG signal [10–12]. The conventional techniques based on Fourier analysis (e.g., IIR filters) are widely used for EMG-based filtering. However, Fourier analysis is purely based on predefined basis functions, which not only reduces the noise but also attenuate the EMG signal. As an alternative to the usual Fourier transform method, wavelet analysis is also popularised due to its advantages in terms of the time–frequency representation [13–16]. The wavelet-based approaches, however, are also suboptimal because the pre-selected wavelet function is often not suitable for matching the natural property of an EMG signal. Previous studies have also introduced the Empirical Mode Decomposition (EMD) approach to handle EMG signals [17]. Instead of those reported in literatures [18], the EMD is a fully data-driven adaptive time–frequency analysis method, and offers no prior assumption through the overall data processing procedure [19–22].

The EMD algorithm was put forward by Huang et al. and provided the most successful results for the decomposition and time–frequency analysis of non-stationary signals. It is given as a sifting process that decomposes a signal into a finite set of intrinsic mode functions (IMFs), amplitude- and/or frequency-modulated components representing its inherent oscillatory modes. Adriano et al. first employed this technique to filter EMG signals in background activity attenuation [17]. However, the first version of EMD was only used for a single-channel EMG, and did not focus on the accuracy of the decomposed subfrequency bands. In order to alleviate this problem, the Ensemble EMD (EEMD), an adaptive dyadic filter bank, was introduced. This method can effectively eliminate the mode-mixing and physically produce more unique frequency levels. The literature shows that several studies have investigated the de-noising performance for EMG signals using the EEMD algorithm [23]. However, such single-channel based EMD algorithms cannot be directly applied into the multiple-channel EMG signal processing [24]. Moreover, the EMD or EEMD algorithms cannot guarantee the equality of the number of decomposed IMFs across multichannels, and may lead to subsequent EMG-based analyses being physically meaningless. Accordingly, the multivariate extension of EMD (MEMD) and its noise-assisted analysis method, Noise-Assisted Multivariate EMD (NA-MEMD) have been developed recently to produce the same number of IMFs across all channels thereby facilitating direct multichannel analyses with the consideration of cross-channel interdependence (mode-alignment) and single-channel independence (mode-mixing) [25–30].

EEMD has been extensively applied as an accurate and computationally efficient quantitative analysis for electromyography (EMG) signals. The EEMD algorithm can effectively extract the temporal information of EMG time series. However, few papers examine the temporal and spatial characteristics across multiple muscle groups in relation to multichannel EMG signals. In this study, NA-MEMD is proposed to handle the multichannel EMG signal processing. The performance of the proposed method has been validated by comparing it with EEMD and MEMD. The experimental data was obtained from the Center for Machine Learning and Intelligent Systems, University of California Irvine (UCI). The data was donated by the Nueva Granada Military University and the Technopark node Manizales in Colombia. Three criterions are proposed to assess the decomposition performance, (1) the number of intrinsic mode functions; (2) mode-alignment (common frequency scales in the same indexed IMFs across different channels) for the cross-channel interdependence; (3) mode-mixing (a single IMF containing multiple scales and/or a single scale residing in multiple IMFs) for the single-channel independence. Results indicate that both MEMD and NA-MEMD methods (except EEMD) can guarantee equal numbers of IMFs. Specifically, for mode-alignment and mode-mixing, NA-MEMD is optimal compared to MEMD and EEMD, and MEMD is merely better than EEMD.

## Experiments

The experimental data was obtained from the Center for Machine Learning and Intelligent Systems, University of California Irvine (UCI). The data was donated by the Nueva Granada Military University and the Technopark node Manizales in Colombia. UCI consented to cite these datasets in publications [31]. This work is also approved by the Institution Research Ethics Board of University of Electronic Science and Technology of China (UESTC). The databases of 11 male subjects from the healthy group are taken into the study. The subjects undergo three exercise programs associated with the knee joint, leg extension from a sitting position (sitting), flexion of the leg up (standing), and gait (walking), while four electrodes are placed on biceps femoris (BF), vastus medialis (VM), rectus femoris (RF), and semitendinosus (ST). The goniometer is also used to record the angle of the knee joint during the exercise programs. Each subject is asked to perform these exercise programs once, and each exercise program contains approximately five motion repetitions. The period time of motion is about 4 s, 2 s for motion and 2 s for rest.

## Methods

### EEMD

The Empirical Mode Decomposition (EMD) is a fully data-driven and adaptive time–frequency analysis method. It describes a signal as a linear combination of a finite set of intrinsic mode functions (IMFs) and a residual signal. The mathematic representation of EMD can be depicted as

1 $$\begin{aligned} x(t) = \sum _{m=1}^M{c_m(t)}+r(t) \end{aligned}$$

where *x*(*t*) is an original signal, $c_m(t)$ and *r*(*t*) represent the $m^{th}$ IMF and the residual assumed as the $(M+1)^{th}$ IMF, respectively. These resultant IMFs, ${c_m(t)}^M_{m=1}$, are sequentially extracted from the original signal by an iteration algorithm called the sifting processing [19]. For the EMD-based sifting process, the local maxima and minima of *x*(*t*) are first identified, and the upper (lower) envelope is constructed by fitting the local maxima (minima) into a cubic-spline curve. The averaged curve of upper and lower envelopes is then intended to update *x*(*t*) by subtracting it from *x*(*t*). This sifting process will be iteratively executed until each IMF can be determined, while two stoppage criterions should be satisfied, i.e., (*a*) the number of zero crossings and the number of extrema (inclusive of the total number of the local maxima and minima) should not differ by more than one; (*b*) the average value of the upper envelope and the lower envelope through the overall data should be zero. After repeating the above sifting process, all IMFs ${c_m(t)}^M_{m=1}$ are obtained when the residual *r*(*t*) becomes a monotonic function.

For EEMD, the extra white Gaussian noises (WGNs) *w*(*t*) are added with the original signal *x*(*t*) to obtain an ensemble of noise-assisted signal *s*(*t*), i.e., $s(t)=x(t)+w(t)$, and the ensemble signal is decomposed by using the EMD algorithm. This single noise-added procedure is then repeatedly executed, and for each iteration the different realization of white noise $w_n (t)$ is given where $n=1,2,\ldots N$ representing the number of iterations that is set to 50 in this study. The final IMFs can be calculated by averaging the same indexed IMFs of the decomposition. The EEMD algorithm is provided as follows [29]:

1. Input signal, *x*(*t*);
2. Generate $\bar{x}_n(t)= x(t) + w_n(t)$ for $n=1,2,\ldots N$, where $w_n(t)$ ($n=1,2,\ldots N$) are N different realizations of WGN;
3. Identify all local extrema of $\bar{x}_n(t)$;
4. Find lower and upper envelopes, $e_l^n (t)$ and $e_u^n (t)$, which interpolate all local minima and maxima, respectively;
5. Calculate the local mean, $\bar{m}^n(t) = \frac{1}{2}(e_l^n(t)+ e_u^n(t))$;
6. Subtract the local mean from $\bar{x}_n(t)$, $c_m^n(t)=\bar{x}_n(t)-\bar{m}^n(t)$ (*n* is the index number of IMF);
7. Let $\bar{x}_n(t)=c_m^n(t)$ and go to step 3); repeat until $c_m^n (t)$ becomes IMFs;
8. Average the corresponding IMFs from the whole ensemble to obtain the averaged IMFs; for instance, the $m^{th}$ IMF can be obtained by using $\bar{c}_m(t)=\frac{1}{N}(\sum _{n=1}^N c_m^n(t))$.

### MEMD

Rehman and Mandic developed multivariate empirical mode decomposition (MEMD), which is a natural extension of the original EMD/EEMD. In MEMD, the multiple-channel EMGs should be first projected into n-dimensional spaces based on low discrepancy Hammersley sequence. The projections along different directions in multidimensional spaces represent the amplitudes of EMGs across four channels. The extrema are interpolated via cubic-spline interpolation in order to obtain the subenvelopes ${e_{\theta _v}(t)}_{v=1}^V$. Those sub-envelopes are then averaged to obtain a local mean of a multiple-channel EMG signal, M(t). Then, the first IMF can be extracted by subtracting the local mean from the input channels. The outline of MEMD algorithm is presented as follows [25]:

1. Choose a suitable point set for sampling a $(p-1)$ sphere;
2. Calculate a projection, $w_{\theta _v}(t)$, of N-channel input signals $x^N(t)$ (N = 4) along the direction vector $d_{\theta _v}$, for all v (the whole set of direction vectors), giving ${w_{\theta _v}(t)}^V_{v=1}$ as the set of projections;
3. Find the time instant ${t_{\theta _v}(t)}^V_{v=1}$ corresponding to the maxima of the set of projected signal ${w_{\theta _v}(t)}^V_{v=1}$;
4. Interpolate $[t_{\theta _v}(t),x^N(t_{\theta _v})]$ to obtain multivariate envelope curve ${e_{\theta _v}(t)}^V_{v=1}$;
5. For a set of V direction vectors, the mean *M*(*t*) of the envelope curve is computed as $M(t)=\frac{1}{V}(\sum _{v=1}^V e_{\theta _v}(t))$;
6. Let $c^N(t)= x^N(t)-M(t)$. If $c^N(t)$ fulfills the stopping criterion for a multivariate IMFs, apply the above procedure to $x^N(t)-c^N(t)$; otherwise, apply it to $c^N(t)$.

Different with EEMD, the sifting process is followed by

2 $$\begin{aligned} \frac{M(t)}{e(t)}<\gamma \end{aligned}$$

where *e*(*t*) is the bias function defined by $e(t)=\frac{1}{N}\sum _{n-1}^N\mid c^n(t)-M(t)\mid$, and the threshold value $\gamma$ was set to 0.2 based on the EMG signals during lower-limb exercises. The sifting process will be continued if Eq. (2) is satisfied.

### NA-MEMD

**Fig. 1:**
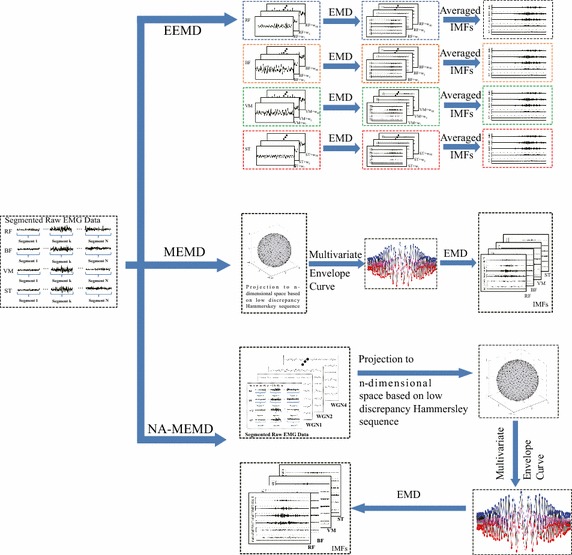
The schematic diagram for EEMD, MEMD, and NA-MEMD

The Noise-Assisted multivariate empirical mode decomposition (NA-MEMD) was also introduced by Rehman and Mandic. This signal processing work exploits the dyadic filter properties of EMD and MEMD. Additionally, it also applies the noise assisted analysis method into MEMD, a dyadic filter bank on each channel while adding certain multidimensional WGNs together with the original signals which are decomposed by using MEMD. More specifically, K-channel ($K \ge 1$) uncorrelated WGNs time series of the same length with that of the M-channel EMGs ($M = 4$) are randomly separately created. Then, a new input multichannel signal is constructed by adding the original EMGs with the noise channel, the resulting $(M+K)-channel$ multivariate signal. Considering the decomposition of the constructed signal, the remaining procedures are strictly followed by those of MEMD [32]. Figure 3 outlines the processing procedure of NA-MEMD. The effects of the number of noise channels and noise power in NA-MEMD are discussed in [33]. In this study, the average STD based on all EMG channels is selected as the residual noise power, and the number of noise channel is set to four. The schematic diagram for methods of EEMD, MEMD, and NA-MEMD is presented in Fig. 1.

### Data preprocessing and evaluation criterions

#### Data preprocessing

**Fig. 2:**
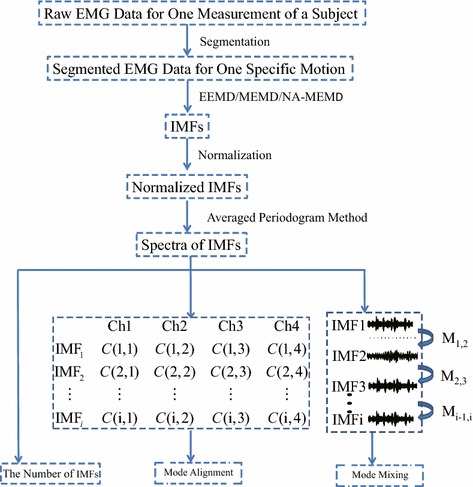
The schematic diagram for evaluation criterions

The raw EMG data measured from each subject were first segmented. The period of the exercise motion was reserved and labeled. The approaches of EEMD, MEMD, and NA-MEMD are then used to decompose these segmented EMG data, by which the decomposed IMFs are obtained via three methods, and then normalized by using standard deviation. Based on each single normalized IMF data, the alignment of IMF based frequency bands in cases of EEMD, MEMD, and NA-MEMD is estimated by the spectra analysis. The last step is to evaluate three criterions, the number of IMFs (indicating cross-channel interdependence), mode-alignment (estimating the alignment of the frequency bands of the same-index IMFs across channels), and mode-mixing (estimating the similarity of the frequency bands of IMFs within a single channel). Figure 2 depicts the schematics for data flow and evaluation criterions, the number of IMFs, mode-alignment, and mode-mixing.

#### Mode-alignment

The mode-alignment effect indicates the common frequency scales in the same indexed IMFs across different channels. This effect would numerically analyse the correlations of frequency scales for each component of the EMG channels, and take advantages of comparatively analysing the frequency similarity of the same-indexed IMFs across channels. In order to obtain this performance, the power spectral density (PSD) of the normalized IMF is first calculated. The PSD correlations between two IMFs are then obtained by $c_{i,j}$, where *i* stands for the *i*th indexed IMF, and *j* is for the number of the channel. The correlation matrix for all IMFs across channels could be expressed as

3 $$\begin{aligned} C_{MA}= \begin{bmatrix} c(1,1)&c(1,2)&\ldots&c(1,j) \\ c(2,1)&c(2,2)&\ldots&c(2,j) \\ \vdots&\vdots&\ddots&\vdots \\ c(i,1)&c(i,2)&\ldots&c(i,j) \end{bmatrix} \end{aligned}$$

The elements in the *i*th row of the correlation matrix are averaged to represent the mode-alignment value in the *i*th indexed IMF.

#### Mode-mixing

The mode-mixing effect describes the overlap of frequency information among the decomposed IMFs within one EMG channel, which would reflect whether or not a single IMF contains multiple scales and/or a single scale resides in multiple IMFs [34]. In this study, we used the following equation to quantitatively describe the mode-mixing effects, $MM_{i,j}$,

4 $$\begin{aligned} MM_{i,j}=\frac{max([f_{2i},f_{8i}]\cap [f_{2j},f_{8j}]) -min([f_{2i},f_{8i}]\cap [f_{2j},f_{8j}])}{min\{D_i,D_j\}} \end{aligned}$$

where $f_{2i}$, $f_{8i}$, and $D_i$ are the PSD of the ith indexed IMF. $f_2$, $f_8$ are the frequencies at which 20 and 80% of the energy of an IMF are reached, respectively. $D_i$ is the difference between $f_{2i}$ and $f_{8i}$. Based on Eq. (4), the mode-mixing effect for a single EMG channel could be calculated as

5 $$\begin{aligned} \tilde{M}=\sum _{i=1}^{I-1} MM_{i,i+1} \end{aligned}$$

where I is the total number of IMFs.

## Results

**Fig. 3:**
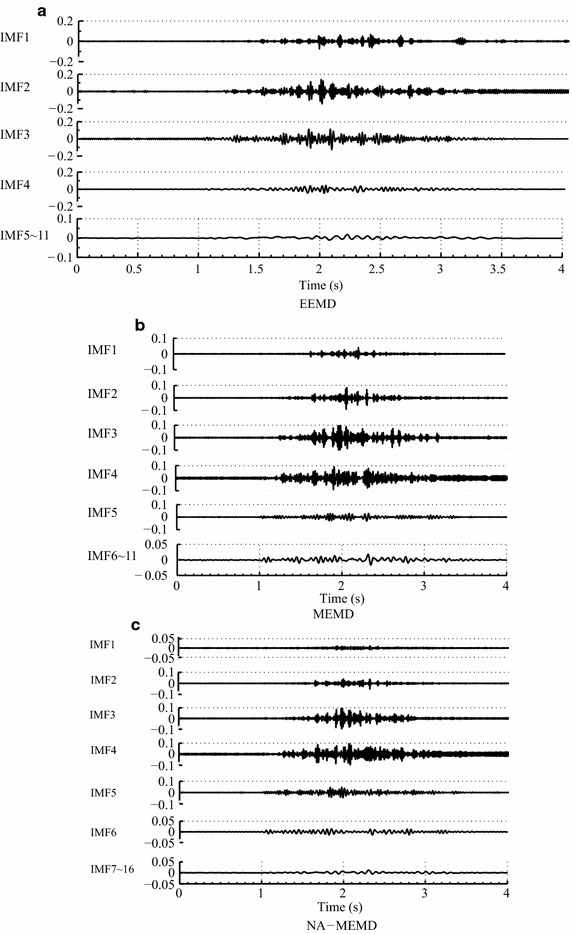
The decomposition result in the vastus medialis muscle group for three exercise programs (sitting, standing, and walking) for EEMD, MEMD and NA-MEMD. **a** EEMD; **b** MEMD; **c** NA-MEMD

Figure 3 shows an example of the decomposition result in the vastus medialis muscle group for three exercise programs (sitting, standing, and walking) for EEMD, MEMD and NA-MEMD. Since the most predominant energy for an EMG signal is approximately between 20 and 500 Hz [5], the decomposed components that have lower subfrequency bands than 20 Hz are synthesized together (from the 5th to 11th IMFs, the 6th to 16th IMFs, and the 7th to 16th IMFs for EEMD, MEMD, and NA-MEMD, respectively).

### Spectra analysis

**Fig. 4:**
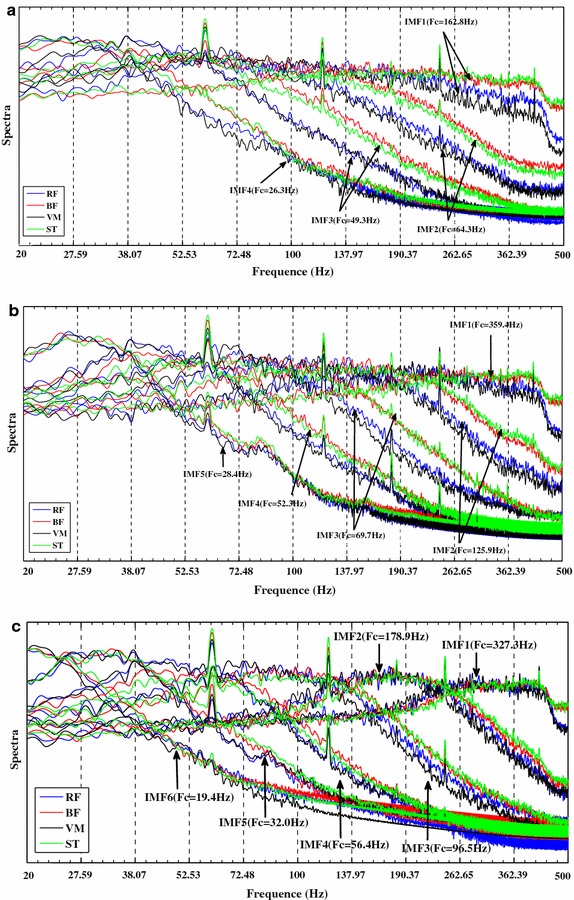
Spectra of normalized IMFs (IMF1-IMF4 for EEMD, IMF1-IMF5 for MEMD, and IMF1-IMF6 for NA-MEMD) obtained from four-channel EMG signals (RF, BF, VM, and ST) via EEMD (**a**), MEMD (**b**) and NA-MEMD (**c**). Overlapping of the frequency bands corresponding to the same-index IMFs is more prominent in the cases of MEMD and NA-MEMD but the NA-MEMD bands clearly show much better alignment

In order to analyse IMF based frequency components produced by EEMD, MEMD, and NA-MEMD, the decomposed IMFs, specifically representing one of exercise motions, are first normalized. These IMFs are then utilized for the analysis of spectra. Figure 4 indicates the spectra results of IMFs for three exercise motions decomposed by EEMD, MEMD, and NA-MEMD. In this study, we only focus on the shape of individual spectra in considerations of mode-alignment and mode-mixing. The alignment of frequency bands of the same-index IMFs in muscles BF, VM, RF, and ST is closer, the mode-alignment performance is more prominent. The spectra figures only can qualitatively analyze and demonstrate the differences of decomposition by EEMD, MEMD, and NA-MEMD, in which the stabilization of the shape of individual spectra from BF, VM, RF and ST can be observed. Based on these spectra information, the statistical analyses are used to quantitatively estimate the performance of mode-alignment and mode-mixing.

### The number of IMFs

**Table 1:** Statistics results for the number of IMFs by using EEMD, MEMED, and NA-MEMD for four-channel EMG signals related to lower-limb functional activities of daily living

	RF	BF	VM	ST
Sitting				
EMD	12.27 ± 1.20	12.45 ± 1.29	12.09 ± 0.94	12.18 ± 0.75
MEMD	*16.45* ± *0.93*	*16.45* ± *0.93*	*16.45* ± *0.93*	*16.45* ± *0.93*
NA-MEMD	*16.64* ± *0.67*	*16.64* ± *0.67*	*16.64* ± *0.67*	*16.64* ± *0.67*
Standing				
EMD	12.27 ± 1.10	13.00 ± 0.63	12.18 ± 0.87	12.18 ± 1.60
MEMD	*15.64* ± *0.92*	*15.64* ± *0.92*	*15.64* ± *0.92*	*15.64* ± *0.92*
NA-MEMD	*16.55* ± *0.93*	*16.55* ± *0.93*	*16.55* ± *0.93*	*16.55* ± *0.93*
Walking				
EMD	10.73 ± 0.79	10.91 ± 0.54	10.54 ± 0.67	10.64 ± 0.67
MEMD	*14.27* ± *0.47*	*14.27* ± *0.47*	*14.27* ± *0.47*	*14.27* ± *0.47*
NA-MEMD	*15.09* ± *0.30*	*15.09* ± *0.30*	*15.09* ± *0.30*	*15.09* ± *0.30*

Italic values are statistically significant

A statistical survey is also taken by investigating the EMG signals of muscle groups RF, BR, VM, and ST to sitting, standing, and walking exercises for all subjects. The averaged number of IMFs for each muscle is shown in Table 1. It has been clearly shown that MEMD and NA-MEMD could guarantee the equal number of IMFs across EMG different channels. In addition, the number of IMFs via MEMD and NA-MEMD have a larger amount compared to those of EEMD, indicating that more details of EMG frequency components can be obtains based on MEMD and NA-MEMD results.

### Mode-alignment

**Table 2:** Statistics results for the mode-alignment effects based on the motion segmentations of four-channel EMG signals from all subjects related to lower-limb functional activities of daily living

Subject	Sitting			Standing			Walking		
	EMD	MEMD	NA-MEMD	EMD	MEMD	NA-MEMD	EMD	MEMD	NA-MEMD
1	0.73	0.78	*0.84*	0.65	0.74	*0.76*	*0.82*	0.79	0.78
2	0.73	0.78	*0.84*	0.65	0.74	*0.76*	0.62	0.78	*0.79*
3	0.72	0.82	*0.88*	0.68	0.80	*0.80*	0.67	0.80	*0.83*
4	0.68	0.78	*0.80*	*0.77*	0.75	0.76	0.71	*0.78*	0.76
5	*0.76*	0.70	*0.76*	0.72	0.80	*0.82*	0.51	0.71	*0.76*
6	0.66	0.64	*0.75*	0.80	*0.81*	0.80	0.81	*0.84*	0.81
7	0.43	*0.80*	0.77	0.68	0.88	*0.89*	0.61	0.66	*0.84*
8	0.54	0.82	*0.86*	0.69	0.88	*0.89*	0.50	0.55	*0.79*
9	0.71	0.87	*0.87*	0.68	0.90	*0.92*	0.64	*0.80*	0.79
10	0.59	0.90	*0.91*	0.60	0.82	*0.89*	0.76	0.77	*0.79*
11	0.81	0.83	*0.86*	0.62	0.86	*0.88*	0.34	0.57	*0.72*
Mean	0.67	0.79	*0.83*	0.69	0.82	*0.83*	0.64	0.73	*0.79*
STD	0.11	0.07	*0.05*	0.06	0.06	*0.06*	0.14	0.10	*0.03*

Italic values are statistically significant

**Table 3:** Results of two-way ANOVA (exercise programs $\times$ methods) for mode-alignment

Source of variation	Sum of squares	Degree of freedom	Mean square	F-statistic	p
Exercise programs	0.063	2	0.031	2.257	0.131
Methods	0.417	2	0.372	32.022**	0.000
Exercise	0.006	4	0.003	0.342	0.717
Programs × methods					

** p < 0.01 change within the methods among EEMD, MEMD, and NA-MEMD

**Fig. 5:**
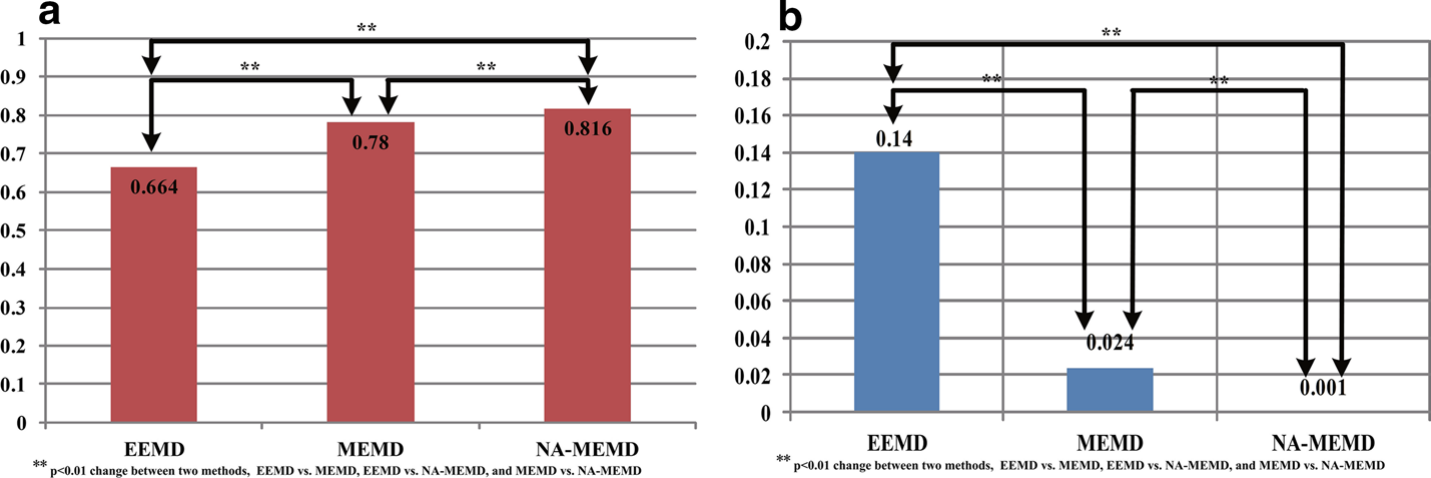
One-way repeated measures ANOVA for EEMD versus MEMD, EEMD versus NA-MEMD, and MEMD versus NA-MEMD. The *left subfigure* indicates the comparative results for mode-alignment. The *right one* indicates those for mode-mixing

In order to statistically analyse the mode-alignment performance for multiple-channel EMGs, the correlation matrixes based on the motion segmentations of four-channel EMG signals from all subjects in three exercise programs are calculated. The IMFs with the subfrequency energy less than 20Hz are removed as it contains much noise and has a low signal-to-noise ratio. The mode alignment effects of decomposed IMFs of four-channel EMG data obtained from the health group are identified in Table 2. Based on these results, two-way analysis of variance (ANOVA) is used to examine the influence of exercise programs (i.e., sitting, standing, and walking) and methods (i.e., EEMD, MEMD, and NA-MEMD) on the performance index of mode-alignment (Table 3). The assessment results show that the methods have a significant main effect ($p<0.01$), and no interaction between exercise programs and methods ($p>0.05$). The statistic analysis with no interaction effect confirms that the three types of exercise programs equally represent the characteristic of functional activities of daily living. In order to further evaluate the difference among methods, the mode-alignment values in three exercise programs for each subject are averaged, and then the One-way Repeated Measures ANOVA is used to compare the mode alignment of IMFs by EEMD, MEMD, and NA-MEMD. It is clear that there is a significant difference among three methods ($F=32.022$, $p=0.000$). By using the Least Significant Difference (LSD), the mode-alignment effect of NA-MEMD is the best among three methods, and the effect of MEMD is merely better than that of EEMD (Fig. 5).

### Mode-mixing

**Table 4:** Statistics results for the mode-mixing effects based on the motion segmentations of four-channel EMG signals from all subjects related to lower-limb functional activities of daily living

Subject	Sitting			Standing			Walking		
	EMD	MEMD	NA-MEMD	EMD	MEMD	NA-MEMD	EMD	MEMD	NA-MEMD
1	0.09	0.067	*0*	0.14	0.01	*0*	0.03	0.01	*0*
2	0.09	0.067	*0*	0.14	0.01	*0*	0.05	*0*	0.01
3	0.12	*0*	*0*	0.09	0.02	*0*	0.14	*0*	*0*
4	0.09	0.03	0.04	0.14	0.02	*0*	0.11	0.04	*0*
5	0.18	0.05	*0*	0.15	0.02	*0*	0.21	0.03	*0.0014*
6	0.27	0.06	*0*	0.16	*0*	*0*	0.06	*0*	*0*
7	0.10	*0*	*0*	0.08	*0*	*0*	0.33	0.10	*0*
8	0.18	0.01	*0*	0.12	*0*	*0*	0.20	0.04	*0*
9	0.17	*0*	*0*	0.10	*0*	*0*	0.11	*0*	*0*
10	0.13	*0*	*0*	0.24	0.02	*0*	0.15	0.01	*0*
11	0.13	0.04	*0*	0.08	*0*	*0*	0.27	0.11	*0.02*
Mean	0.14	0.03	*0*	0.13	0.01	*0*	0.15	0.03	*0.003*
STD	0.06	0.03	*0.001*	0.05	0.01	*0*	0.09	0.04	*0.005*

Italic values are statistically significant

In this study, we also investigate the mode-mixing effect based on each muscle channel by using Eqs. (4) and (5). In order to avoid the effects of inter-subject variability, the mode-mixing effects from all subjects are investigated. For each single subject, the decomposed IMFs for each muscle channel with a central frequency of the spectrum less than 20 Hz are also removed. The mode-alignment $MM_{i,i+1}$ $(\text{i}=1, 2,\ldots,\text{I})$from the remaining IMFs for each muscle channel are calculated. The mode-alignment effect for each muscle channel $\tilde{M}$ is then obtained by averaging the set of $MM_{i,i+1}$ $(\text{i}=1, 2,\ldots,\text{I}).$Following this procedure, the mode-mixing effects of four EMG channels for three exercise programs are provided in Table 4.

**Table 5:** Results of two-way ANOVA (exercise programs $\times$ methods) for mode-mixing

Source of variation	Sum of squares	Degree of freedom	Mean square	F-statistic	p
Exercise programs	0.004	1.344	0.003	0.644	0.481
Methods	0.368	1.234	0.299	138.687**	0.000
Exercise	0.002	1.708	0.001	0.307	0.706
Programs × methods					

$$^{**}\,p<0.01$$ change within the methods among EEMD, MEMD, and NA-MEMD

In a similar way, the influence of exercise programs and methods on mode-mixing is first quantitatively analyzed through two-way ANOVA. Table 5 indicates the main effect of methods ($p<0.01$) as well as no interaction effect between two factors ($p=0.706$). The mode-mixing values in the three exercise programs of each subject are averaged, and the averaged values are applied to test the influence of methods on the performance of mode mixing by using One-way Repeated Measures ANOVA and LSD. It can be seen from Fig. 5 that there are significant differences between two methods (EEMD vs. MEMD, EEMD vs. NA-MEMD, and MEMD vs. NA-MEMD) for the performance of mode mixing of decomposed IMFs. The NA-MEMD achieves the best mode mixing performance compared to EEMD and MEMD, while MEMD far outperforms that of EEMD.

## Discussion

The objective of this study is to evaluate a superior solution for the preprocessing of multichannel EMG signals as well as for the analysing of the IMF based frequency components related to multiple muscle groups. The muscle coordination often occurs in human motions, which is not only indicated by multichannel EMG signals, but also conducted by neuromuscular patterns [35]. Generally, the neuromuscular pattern is intrinsic for the specific exercise motions. Therefore, the single-channel-based analyses for the observation of the nervous system and its corresponding muscle contraction are not sufficient.

Additionally, similar with the ECG lead system [36], it is also desirable to develop the EMG lead system in which the behaviors of motor units can be represented as a set of statistically independent sources.

The human exercise is often supported by multiple relative muscles. For example, the muscle groups of BF, VM, RF and ST are the muscles related to the knee movement such as standing, sitting, and walking. Hence, the use of the so-called four-lead system (the leads placed on muscles BF, VM, RF and ST) would well indicate the overall neuromuscular patterns, which are further controlled by the human brain activity. Moreover, it is a natural way to simultaneously decompose the multichannel EMG signals and analyse the subfrequency bands of multichannel EMG signals.

Although previous literatures have reported the successful applications of EMD/EEMD in the single-channel EMGs [17], these approaches cannot solve the critical problem about the fusion and analysis of multichannel EMG signals [30, 34, 37]. Therefore, EEMD, MEMD and NA-MEMD have been investigated in this study for the decomposition performance of four knee muscle groups associated with standing, sitting, and walking. Three criterions (the number of IMFs, mode-alignment and mode-mixing) are employed to quantitatively depict the decomposition efficiency.

It has been confirmed that both MEMD and NA-MEMD (exclusive of EEMD) could provide an equal number of IMFs across EMG different channels. If the number of IMFs is unequal, then the decomposed subfrequency signals cannot be directly applied for the subsequent study. This also leads to the similar oscillation modes appearing in multiple IMFs (Fig. 3a).

The mode-alignment effect focuses on the cross-channel dependence. In order to compare the same indexed IMFs among muscle channels, a similar subfrequency band of the same indexed IMFs should also be observed. The statistics show that there is a significant difference among three methods (F = 32.022, p = 0.000). Moreover, the effect of NA-MEMD is the best among three methods. In addition, the effect of MEMD is better than that of EEMD.

For the assessment of the mode-mixing effect, there are significant differences between two methods (EEMD vs. MEMD, EEMD vs. NA-MEMD, and MEMD vs. NA-MEMD). Specifically, NA-MEMD achieves the best mode-mixing performance compared to EEMD and MEMD, and the effect of MEMD outperforms that of EEMD.

## Limitation

The experimental data in this study was obtained from the Center for Machine Learning and Intelligent Systems, UCI. The data was donated by the Nueva Granada Military University and the Technopark node Manizales in Colombia. The physical characteristics of the participants were not recorded in the datasets. There also was no information about the prior nutritional intake, physical activity and environment conditions before all participants engaged in the experimental sessions. In addition, as the exercise programs (i.e., sitting, standing, and walking) are only taken from one measurement, the intra-subject variability such as random errors may not be avoided. The experiment description contained in the datasets did not clearly specify the location of electrodes placed on muscles BF, VM, RF, and ST.

## Conclusions

This study proposed the noise-assisted multivariate empirical mode decomposition (NA-MEMD) approach for the preprocessing of multiple channel EMG signals, by which the temporal and spatial characteristics across multiple muscle groups can be quantitatively depicted. The four muscle groups of BF, VM, RF, and ST associated with lower limb exercises (sitting, standing, and walking) of 11 healthy subjects were utilised for the assessment of the EMD-based approaches. A comparative study was provided by assessing the NA-MEMD with Ensemble Empirical Mode Decomposition (EEMD), and Multivariate EMD (MEMD). Three criterions were used to assess the comparative outcomes, i.e., the number of intrinsic mode functions (IMFs), mode-alignment and mode-mixing. The results indicated that the current EMD-based approach of using EEMD was suboptimal for multichannel EMG signals due to its poor performance in relation to the three criterions. When compared with MEMD and NA-MEMD, both approaches with data from lower limb EMG signals would guarantee an equal number of IMFs across channels. In addition, the statistical results showed that both the mode-alignment and mode-mixing effects of NA-MEMD were superior to those of MEMD. This finding implied that NA-MEMD is effective for simultaneously analysing IMFs based frequency bands. It has a vital clinical implication in terms of exploring the neuromuscular patterns that enable the coordination of multiple muscle groups for the purposes of performing daily activities.

## Abbreviations

BF: biceps femoris
EMG: electromyography
ECG: electrocardiography
EMD: empirical mode decomposition
EMG: electromyography
EEMD: ensemble empirical mode decomposition
GM: goniometry
IIR: infinite impulse response
IMF: intrinsic mode functions
MEMD: multivariate empirical mode decomposition
NA-MEMD: noise-assisted multivariate empirical mode decomposition
PSD: power spectral density
RF: rectus femoris
ST: semitendinosus
STD: standard deviation
VM: vastus medialis
WGN: white Gaussian noise
